# The Comparison of Honey Enriched with Laboratory Fermented Pollen vs. Natural Bee Bread in Terms of Nutritional and Antioxidant Properties, Protein In Vitro Bioaccessibility, and Its Genoprotective Effect in Yeast Cells

**DOI:** 10.3390/molecules28155851

**Published:** 2023-08-03

**Authors:** Michał Miłek, Mateusz Mołoń, Patrycja Kielar, Ewelina Sidor, Aleksandra Bocian, Katarzyna Marciniak-Lukasiak, Anna Pasternakiewicz, Małgorzata Dżugan

**Affiliations:** 1 Department of Chemistry and Food Toxicology, Institute of Food Technology and Nutrition, University of Rzeszow, Ćwiklińskiej 1a St., 35-601 Rzeszów, Poland; ewelina.sidor.dokt@gmail.com (E.S.); apasternakiewicz@ur.edu.pl (A.P.); mdzugan@ur.edu.pl (M.D.); 2 Institute of Biology, University of Rzeszów, 35-601 Rzeszów, Poland; mmolon@ur.edu.pl (M.M.); kielar.patrycja98@gmail.com (P.K.); 3 Doctoral School, University of Rzeszów, Rejtana 16c, 35-959 Rzeszów, Poland; 4 Department of Biotechnology and Bioinformatics, Faculty of Chemistry, Rzeszów University of Technology, Powstańców Warszawy 6, 35-959 Rzeszow, Poland; bocian@prz.edu.pl; 5 Institute of Food Sciences, Faculty of Food Assessment and Technology, Warsaw University of Life Sciences (WULS-SGGW), Nowoursynowska 159c, 02-776 Warsaw, Poland; katarzyna_marciniak_lukasiak@sggw.edu.pl

**Keywords:** honey, bee pollen, bee bread, pollen fermentation, protein, bioaccessibility, antioxidant activity, genoprotection

## Abstract

The aim of the study was to compare the nutritional value and bioactivity of honey enriched with a 10% addition of natural bee bread and its substitutes obtained as a result of laboratory fermentation of bee pollen. Physicochemical parameters, antioxidant properties, as well as the bioaccessibility of proteins using an in vitro static digestion model were analyzed. The bioactivity of the obtained enriched honeys was tested using the yeast model. The research indicates the similarity of honeys with the addition of “artificial bee bread” to honey with natural ones. During in vitro digestion, good bioaccessibility of the protein from the tested products was demonstrated. The ability of the products to protect yeast cells against hydrogen superoxide-induced oxidative stress was demonstrated using a qualitative spot test, which was stronger in the case of enriched honey than in pure rapeseed control honey. Significant inhibition of the growth of both strains of yeast exposed to bee pollen-enriched honeys was also demonstrated. Furthermore, all tested samples showed significant genoprotective activity against the genotoxic effect of zeocin and the reduction of the number of DNA double-strand breaks by a minimum of 70% was observed.

## 1. Introduction

The sensory quality and health-promoting properties of honey can be shaped by various types of additives, most often introduced to it during the creaming process [1,2,3,4]. The process of creaming honey consists of obtaining it in a crystallized form under controlled conditions, thanks to which a smooth consistency is obtained, suitable for mixing with various additives. Although currently honeys with herbs and dried fruits are very popular on the market, the first characterized products of this type were created as a combination of honey with other products of bee origin: propolis, bee pollen, royal jelly, and bee bread [1]. The combination of honey with these products, known for their antioxidant, antimicrobial, anti-inflammatory, and other beneficial properties, allows the creation of new products with functional properties [5].

Bee bread, a form of pollen processed by bees, has nutritional, antioxidant, hypolipidemic, hepatoprotective, anticancer, antimicrobial, and antiviral properties due to its high content of protein, sugars, and lipids [6,7,8]. Due to its unattractive sensory properties, mixing it with honey has been well-known for a long time and it was traditionally used by beekeepers practices which allow the introduction of bee bread to the diet [4]. The addition of bee bread to honey is usually high, most often 10–20% (*w*/*w*), but the literature gives examples of 60% share [1]. Earlier studies of honeys with the addition of bee bread showed that it is a product with high antioxidant activity, containing an increased content of phenolic acids and flavonoids [9]. A significant effect of enrichment with bee bread was also shown by the physicochemical parameters of honey and the content of minerals, which means that such a product can be a valuable source of micro and macroelements in the human diet [10], remembering the crucial role of some microelements (i.e., Zn and Fe) present in some wild species that are important to man [11,12]. Bee bread is also rich in protein (approx. 15–28%) [7], which means that enriching honey with this product would make it a source of protein and exogenous amino acids. Due to the limited production of bee bread in bee colonies, attempts are made to imitate this natural process by conducting controlled fermentation of pollen in laboratory conditions [13,14,15,16,17]. The expected effect of this approach is to increase the supply of bee bread (the so-called “artificial bee bread”), which can also be used as an addition to honey. So far, such products are not present on the market, and no scientific studies have compared them with honey enriched with natural bee bread.

All bee products show a wide range of biological activity. In addition to well-researched properties, there are some very valuable and less explored areas [18,19]. The genoprotective effect, i.e., the protection of the genetic material of cells against damage caused by various physical and chemical factors, is one of the new fields of research on natural products [20,21]. Such an effect so far has been demonstrated for many bee products, including honey [22], bee pollen [23], royal jelly [24], and propolis [25]. However, bee bread, alone or in combination with honey, has not been studied for its genoprotective effect. The synergy of the genoprotective effect of honey, propolis, and royal jelly has been demonstrated previously [26], and such an effect can also be expected for the combination with bee bread.

The aim of the research was to compare creamed honeys enriched with natural bee bread and its substitute obtained by laboratory fermentation in terms of physicochemical properties, nutritional value, and antioxidant and genoprotective effects.

## 2. Results and Discussion

### 2.1. Physicochemical Evaluation

The produced samples of honey enriched with the addition of natural bedding (two samples of bee bread from different locations, HBB1 and HBB2) and several variants of pollen fermented in laboratory conditions (HFP1-HFP5, description of the samples in Material and Methods section) were subjected to comparative analyses, also concerning the initial honey used for creaming (H). The results of selected physicochemical parameters tested for pure honey and enriched samples are compared in Table 1.

Physicochemical parameters are important features determining the commercial quality of honey, hence their study was aimed at determining the impact of the introduced additives on the overall quality of the product following the applicable EU legislation [27]. There has been no observed deterioration in the quality of honey in terms of water content; it does not exceed the required standards of 20% in any case. In the samples with the addition of bee bread and fermented pollen, a decrease in this parameter was observed in comparison with the rapeseed honey used. It can be assumed that the introduced additives bind the water naturally present in the honey, swelling at the same time. Additionally, water activity, a key parameter for susceptibility to microbial deterioration, was determined. Except for the HBB2 sample, a significant reduction in water activity was obtained, which is beneficial for the stability of the product. Moreover, water activity positively correlated with the refractometric moisture content (*r* = 0.702). Water content, and especially water activity, are important parameters indicating the possible susceptibility of honey to fermentation, hence determining the influence of additives on these parameters allows us to confirm that the product is stable and not threatened by processes that deteriorate its quality. A significant increase in electrical conductivity among the tested samples was observed. The value of conductivity for honey is correlated with the content of minerals and organic acids [28]. The value of this parameter for the initial honey was low (0.149 mS/cm), which is typical of rapeseed honey [29]. The introduction of fermented additives caused a multiple increase in conductivity as a result of enrichment with minerals and organic acids derived from bee bread and pollen. The highest increase was noted in the case of HFP4 and HFP5 samples, where the addition of fermented pollen inoculated with a starter culture obtained using milk as a medium was used, which may also have a significant impact on the tested parameters. Habryka et al. [10] showed that the addition of pollen and bee bread proportionally increases the ash content of honey. Literature data indicate that both parameters are also strongly correlated with each other [30]. The greatest change was observed in the acidity of the obtained products. The rapeseed honey used was characterized by low acidity, which proves its good quality and freshness. As a result of the enrichment, the free acidity increased even more than 10 times, which is related to the nature of the additives, which are fermented products and contain high lactic acid levels. The final pH value was the result of the pH of the honey itself and the additives used. Fermented pollen has a higher pH value than bee bread, which is the result of incomplete fermentation in laboratory conditions [17]. Because of this, the pH value of honey enriched with natural bee bread (HBB samples) was lower than for honey with fermented pollen (HFP samples). The determined energy value of honey was typical for this variety, which is consistent with the value declared on the label (333 kcal/100 g). As a result of the addition of bee bread and laboratory-fermented pollen, a significant increase in calorific value (at the level of 2.5 to 5%) was obtained, the highest in the case of pollen fermented with the use of *Lactobacillus acidophilus* (Moro) starter culture grown in milk. The resulting increase in energy value is beneficial from a nutritional point of view.

**Figure 1:**
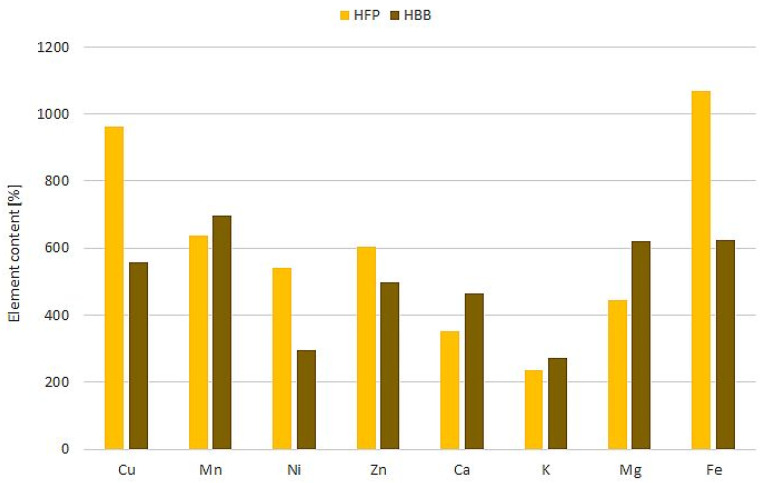
Average percentage increase in the content of the element in honey enriched with natural bee bread (HBB) and fermented pollen (HFP) in relation to rapeseed honey (100%) calculated based on data for raw materials.

Due to the high content of minerals in pollen and bee pollen, it can be expected that mixing honey with such additives will increase the content of elements in honey. Because we had previously examined the mineral composition of rapeseed honey [17], bee pollen, and fermented pollen used for enrichment [31], it was possible to calculate the percentage of the enrichment of honey with selected elements (Figure 1).

According to the estimated results, adding bee bread or fermented pollen to honey can be enriched up to 10 times with selected elements, especially those classified as microelements. Multiple enrichment of honey with macroelements (K, Na, Ca, Mg) was previously confirmed by Habryka et al. [10], who found the dependence of the content of these elements was on the share of the introduced additive, achieving the highest enrichment in magnesium (almost 25 times with a 25% addition of bee pollen and 20 times with a 25% addition of bee bread). In the case of the analyzed microelements (Fe, Zn, Cu, Mn), the same trend was also observed, and the highest degree of enrichment was observed for iron [10]. This element is particularly important for human health, it is involved in many biochemical processes, including oxygen transport through the blood, DNA synthesis, and redox reactions. Iron deficiency is the cause of anemia, hence the search for new sources of high bioavailability of iron in the diet [32]. Thanks to the increase in the content of important macro and microelements, honey enriched with bee bread or its substitute obtained in the laboratory can be a good way to increase the supply of essential elements in the human diet.

### 2.2. Total Phenolic Content and Antioxidant Properties

As an important indicator of biological activity, the total content of phenolic compounds and antioxidant capacity using the DPPH and FRAP methods were determined. The results are shown in Table 2.

The analysis of phenolic compounds’ content and antioxidant properties clearly shows the improvement of honey properties as a result of the introduction of bee bread into it. Rapeseed honey used as a base was characterized by moderate antioxidant properties, typical for honey of this variety [33]. In each case, a significant increase in the assessed properties was noted in relation to the control sample (rapeseed honey). The multiple increases in the content of polyphenols and the antioxidant capacity of honey with introduced bee pollen or laboratory-fermented pollen results directly from the properties of these additives [9,17]. There were no significant differences between the samples used for enrichment, except for the significantly highest content of polyphenols in the case of honey with FP4 (119.77 mg GAE/100 g) and antiradical activity in the case of honey with FP5 (68.40 μmol TE/100 g). This is probably due to the similarity of the additives used for fermented pollen samples, for which the substrate was the same initial bee pollen. HFP4 and HFP5 samples were enriched with pollen fermented by a different procedure, which probably ensures the highest content of bioactive substances compared with other products.

Thanks to the fast rate of crystallization, easy availability, and low price, rapeseed honey is most often used as a base for the preparation of creamed honey with additives, including other bee products. However, in the literature, the results for honey enriched in bee bread are available for multifloral [1,9] and lime honey [5] only. The enrichment of honey with bee bread produced the highest effect among the tested bee products in the study by Juszczak et al. [1]. Kowalski and Makarewicz [5] enriched honey with the addition of 5 to 15% of bee bread and observed a proportional relationship between the increase in activity and the addition of the additive. With the addition of 10%, they obtained an increase in TPC by 17% and antioxidant properties by 32–34%, depending on the analytical method used. They also evaluated honeys with the addition of a combination of bee bread and propolis, obtaining a multiple enhancement of the effects of such complex products. Comparing the results obtained by us, where the enrichment in antioxidants reached seven times in the case of natural bee bread and almost eighteen times in the case of the HFP5 sample, it can be concluded that the matrix used, i.e., the type of honey used for creaming, has a significant impact on the enrichment achieved. In turn, Socha et al. [9] showed the differentiation of the effect achieved depending on the origin of both honey and added bee bread. A multiple increase in honey properties as a result of the introduction of bee pollen was also observed earlier [34].

### 2.3. Protein Content and SDS PAGE Analysis

For the examined honeys, the protein content was determined: Total by the Kjeldahl method and soluble by the Bradford method. The results are summarized in Table 3.

The protein content of nectar honeys is low (between 0.2 and 0.4 g/100 g) [35], as confirmed by the obtained result and the vast majority of the total protein is a soluble protein. In the case of honeys with the addition of natural bee bread and processed pollen, a several-fold increase in protein content was observed, as the introduced additives are rich in them. Furthermore, apart from the soluble protein fraction, the additives used have a lot of non-protein nitrogen, e.g., from free amino acids, whose bee bread as well as fermented pollen contains a rich set [36]. Thanks to this, the obtained products show a high supply of protein valuable for the consumer.

**Figure 2:**
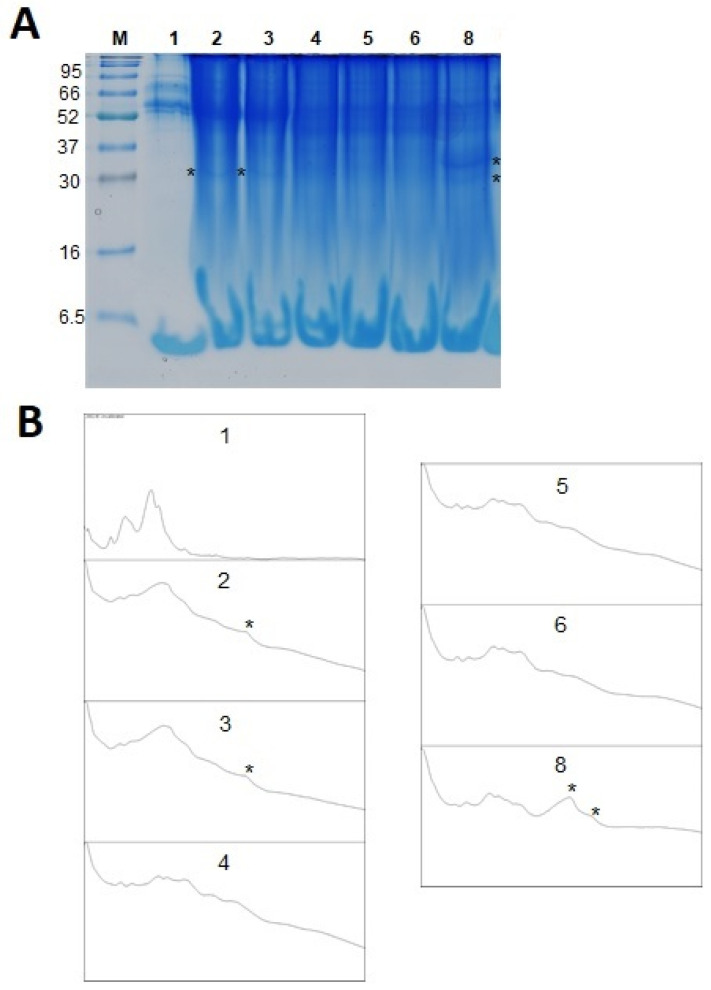
SDS PAGE gel image (**A**) and generated protein profiles (**B**). Tracks description: 1—rapeseed honey (H), 2—HBB1, 3—HBB3, 4—HFP1, 5—HFP2, 6—HFP3, and 8—HFP5. *—additional bands present in enriched honey samples. H—control rapeseed honey, HBB1, HBB2—honey enriched with two samples of natural bee bread, HFP1-HFP5—honey enriched with laboratory-fermented bee pollen FP1-FP5 (symbols as described in Materials and Methods Section 3.2.).

As a result of SDS-PAGE separation (Figure 2), protein profiles of basic and enriched honeys were obtained. The profile of enriched honeys was different, which is visible in the darker color of the entire electrophoretic tracks (Figure 2A). In the control honey, there are four bands with masses in the range of 52–90 kDa, which probably correspond to proteins derived from bees: alpha-glucosidase and Major Royal Jelly Protein [37]. In enriched honeys, darker areas are clearly visible in this area, merging into one area both on gels and graphs. However, additional low-molecular bands are visible at the mass of approximately 30 kDa in samples HBB1 and HBB2 and two additional bands above and below 30 kDa in sample HFP5 (Figure 2A,B). Proteins with masses of approximately 90, 80, 37, 34, and 26 kDa were previously identified in the water-soluble fraction of bee bread [38]. Bands corresponding to proteins with this mass range were also previously observed for bee bread samples of various botanical origins [39]. Proteins with lower molecular weights are probably proteins of plant origin, not present in rapeseed honey. It has previously been shown that bee bread does not differ from bee pollen in terms of protein profile [40]. However, in our earlier study, we observed a much higher band intensity at ca. 65 kDa, attributed to bee alpha-glucosidase [17].

### 2.4. Protein In Vitro Digestibility

**Figure 3:**
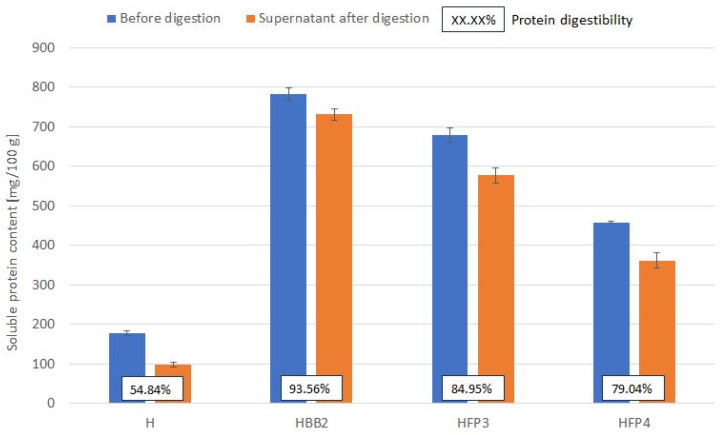
Protein in vitro digestion results. H—control rapeseed honey, HBB2—honey enriched with bee bread, HFP3, HFP4—honey enriched with laboratory-fermented bee pollen FP3 and FP4, respectively (symbols as described in Materials and Methods Section 3.2.).

In vitro protein digestibility was studied using static simulated digestion based on soluble protein content tested by the Bradford method. Analysis of protein digestibility in the prepared honeys showed that rapeseed honey not subjected to digestion contained 0.17 g GAE/100 g of soluble proteins and digestion resulted in protein digestion of 45.13% (Figure 3). Moreover, enriched honeys contained significantly more protein in the undigested fractions from 158 to 341%, with the highest digestibility shown for honey with natural quilts. Protein in honeys with laboratory-fermented pollen was digested to an equally high degree (79.07–84.95%), which may indicate the high bioavailability of the food component analyzed.

Similar protein digestibility results for bee pollen were obtained by Wu et al. [41]—depending on whether the tested pollen was unprocessed or subjected to prior disruption of cell walls, the protein digestibility percentage was up to 64.92% and 87.80%, respectively. Other authors who studied in vitro protein digestion in subsequent steps of the simulated gastrointestinal tract, obtained an increase in digestibility of up to approx. 80% in the small intestine [42]. Moreover, the results obtained for bee pollen and bee bread were comparable [42]. The high protein digestibility demonstrated for bee pollen or bee bread mixed with honey can suggest the disintegrating effect of the honey matrix on pollen cell walls and facilitate the access of digestive enzymes to the internal components of pollen grains.

Other authors, apart from the bioaccessibility of proteins, also studied sugars, lipids, and antioxidant compounds in pollen and bee bread. The digestibility of the fats ranged from 56.01% to 88.18% and was significantly better for pollen that had been previously disrupted [41]. For reducing sugars, the release rate was not so high, ranging from 7.74% to 23.4%, depending on the type of pollen and the pretreatment used [41]. According to the available data, the bioavailability of polyphenols is higher for bee bread than for bee pollen, 31 and 38%, respectively, and a similar relationship occurred for the total flavonoid content. The authors reported a decrease in the antioxidant capacity at the end of in vitro gastrointestinal digestion, both in free radicals scavenging capacity and in reducing power [43]. Higher bioavailability of nutrients and bioactive substances is an often emphasized feature that favors bee bread over bee pollen [44], hence, in terms of the bioavailability of nutrients and bioactive compounds, the addition of bee bread to honey seems to be more justified than crude pollen. In the case of laboratory-fermented pollen, we found slightly lower protein digestibility compared to natural bee bread, the bioaccessibility of other nutrients and bioactive components require further studies.

### 2.5. Biological Activity Using the Yeast Model

**Figure 4:**
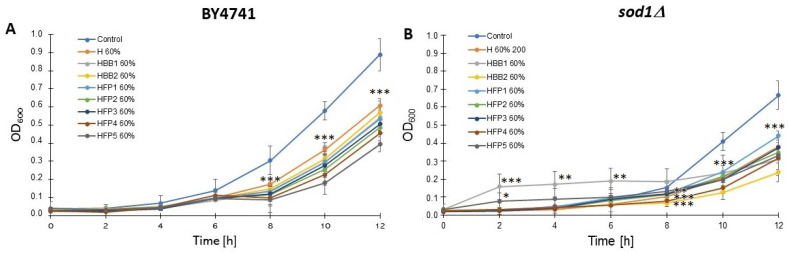
Comparison of growth kinetics of the haploid wild-type yeast strain BY4741 (**A**) and isogenic *sod1Δ* mutant (**B**) treated with the tested honeys. The optical density (OD_600_) of the cultures was measured at different time points for up to 12 h. Error bars represent standard deviations obtained from three independent experiments. Control—negative control without honey, H—control rapeseed honey, HBB1, HBB2—honey enriched with two samples of natural bee bread, HFP1-HFP5—honey enriched with laboratory-fermented bee pollen FP1-FP5 (symbols as described in Materials and Methods Section 3.2.). ANOVA and Dunnett’s post hoc tests were applied and *** *p* < 0.001, ** *p* < 0.01, and * *p* < 0.05 were considered significant. Data represent mean values from three independent experiments.

There are multiple benefits associated with honey, including antioxidants, anti-tumor, anti-inflammatory, and antimicrobial properties. Honey has been demonstrated to inhibit the growth of microorganisms, including yeast. It contains a high sugar concentration that exerts osmotic pressure on microorganisms, causing water to escape through osmosis [45]. However, whether honey solutions inhibit growth or result in cell death has not yet been fully established. Previously, it was shown that solutions of honey and honey with the addition of chokeberry strongly inhibit the growth of wild-type yeast cells. There has been reports that even the slightest dilution of honey can result in the growth of microorganisms, including the yeast *Saccharomyces cerevisiae* [46]. Honey’s antifungal properties also make it an attractive alternative treatment for Candida-related infections [47]. Growth tests on liquid and solid media are a key indicator of the implications of test substances or environmental factors on the cell cycle. In these studies of the bioactivity of honey solutions, we have shown that the tested substances have an inhibitory effect on the growth of yeast cells of both the wild strain and the mutant devoid of Cu, Zn superoxide dismutase. As shown in Figure 4, the tested solutions retard the growth rate of both analyzed yeast strains with different intensities. Interestingly, regardless of the strain, we showed the lowest effect in the case of the HFP1 solution, while the HBB2 and HFP5 solutions showed the most inhibitory effect on the growth rate. Despite the different growth rate of both yeast strains, the growth inhibition effect of exposure to the tested samples was significant and comparable for both.

**Figure 5:**
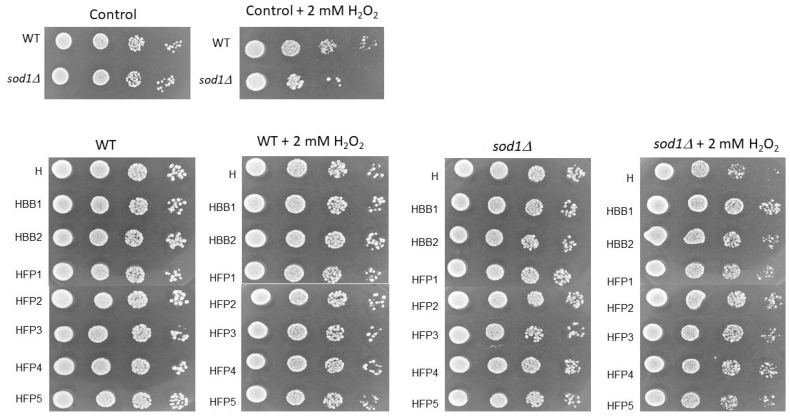
The protective effect of tested honey pre-treatment against H_2_O_2_-induced oxidative stress. Yeast cells were serially diluted as indicated (10^–1^ to 10^–4^) and spotted on YPD media. Control—negative control without honey, 2 mM H_2_O_2_—positive control, H—control rapeseed honey, HBB1, HBB2—honey enriched with two samples of natural bee bread, HFP1-HFP5—honey enriched with laboratory-fermented bee pollen FP1-FP5 (symbols as described in Materials and Methods Section 3.2.).

Honey is one of the oldest known medicinal substances with antioxidant properties. According to some researchers, the usage of honey as a natural product supplement could be considered an adjuvant or therapy in the future [48]. In this study, we used the wild-type strain BY4741 as well as a mutant strain lacking cytosolic superoxide dismutase to check the antioxidant action of tested honeys. At the beginning, cells were pre-incubated in honey solutions for two hours. As a next step, the cells were washed, suspended in a medium, and treated with 2 mM hydrogen peroxide for one hour. After incubation, a series of dilutions of the cells were transferred to rich solid media. As shown in Figure 5, hydrogen peroxide-treated positive control cells grew less than untreated cells (control). Additionally, we demonstrated that honey solution does suppress the negative effects of H_2_O_2_. In this report, we suggest for the first time that the protective properties of bee bread and fermented pollen in honey may be attributed to its strong antioxidant properties. We, therefore, suggest that enriching honey with bee bread or its substitute may have therapeutic applications and support cells in fighting oxidative stress. Further analyses involving eukaryotic models are required to demonstrate the beneficial effects of honey with bee bread in preventing civilization diseases and slowing aging.

**Figure 6:**
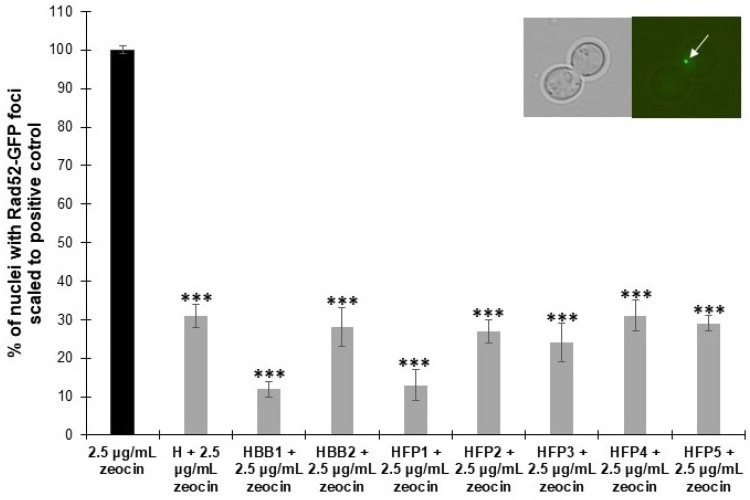
Induction of nuclear Rad52-GFP foci by 2.5 μg/mL zeocin. A sample photo of the Rad52-GFP is presented at the top (the white arrow in the figure’s caption indicates foci). The results represent values for cells tested in two independent experiments (a total of 200 cells). Fluorescence pictures were taken with an Olympus BX-51 microscope equipped with a DP-72 digital camera and cellSens Dimension 4.1 software (1000× *g* magnification). Statistical significances were assessed using ANOVA and Dunnett’s post hoc test (*** *p* < 0.001). Data represent mean values from three independent experiments. H—control rapeseed honey, HBB1, HBB2—honey enriched with two samples of natural bee bread, HFP1-HFP5—honey enriched with laboratory-fermented bee pollen FP1-FP5 (symbols as described in Materials and Methods Section 3.2.).

In addition, we demonstrated that honey solutions act as genome protectors by preventing DNA double-strand breaks. We used Rad52-GFP fusion protein for this purpose. As shown in Figure 6, the use of honey solutions significantly protected yeast cells from the genotoxic effect of zeocin. Zeocin is a copper-chelated glycopeptide antibiotic produced by *Streptomyces verticillus* and causes cell death by intercalating into DNA and cleaving it. This makes it a useful marker for genoprotective activity. Zeocin-induced genotoxicity was reduced by at least 70% for all tested samples compared to the control sample which was not exposed to honey. The strongest effect was noted in the case of honey with natural bee bread (HBB1) and fermented bee pollen (HFP1), which reduced the genotoxicity to approximately 10% compared to the control. Previously, it was shown that coffee also protects cells by preventing DNA double-strand breaks in yeast models [49]. Another method demonstrated the genoprotective effect of bee pollen, especially the extracted lipid fraction, against damage to the genetic material of human lymphocytes caused by doxorubicin [23]. Among other bee products, the greatest genoprotective potential was shown for propolis [25]. Other studies suggest that the protective effect of bee products against DNA damage caused by chemical agents (e.g., benzo(a)pyrene) is related to their antioxidant activity and the polyphenols they contain [26].

## 3. Materials and Methods

### 3.1. Honey, Bee Pollen, and Bee Bread

Rapeseed and multifloral honey were purchased from a locally recognized apiary, guaranteeing high-quality products (49°81′ N, 21°54′ E). The honeys showed typical properties and met the applicable legal regulations (EU). Fresh multifloral bee pollen which dried at 40–42 °C in the form of multicolored grains was purchased from the same local apiary. Two samples of bee bread, with similar organoleptic and qualitative characteristics, from the 2022 beekeeping season were obtained from two local apiaries, located in south-eastern Poland (Subcarpathian Voivodeship) (49°86′ N, 22°56′ E (BB1) and 49°81′ N, 21°54′ E (BB2)).

### 3.2. Bee Pollen Fermentation in Laboratory Conditions

Three samples of fermented pollen (FP1, FP2, and FP3) were prepared as described in Miłek et al. [12]. Additionally, two other samples of fermented pollen with the use of commercial *Lactobacillus acidophilus* (Moro) (LA-5, Ch. Hansen, Hoersholm, Denmark) were produced. The preparation of all samples has been described in Table 4. The starter culture of *L. acidophilus* was prepared by inoculating 10 mL of boiled milk with 1 g of freeze-dried bacteria. The starter culture added to the pollen fermentation was obtained after fermentation for 16 h at 37 °C.

The fermentation was stopped by drying samples (45 °C) to a water content of approx. 10–12% and ground to a powder using a mill (MK-06M, MPM, Milanówek, Poland).

### 3.3. Preparation of Honey Enriched with Bee Bread and Fermented Pollen

The rapeseed honey was completely decrystallized by heating it up to 42 °C for 48 h in a laboratory incubator (SLN 53 STD, Pol Eko, Wodzisław Śląski, Poland). Then, weighed portions of honey were inoculated with crystallized honey (99:1, *w*/*w*), and the portions were mechanically mixed for 60 s four times a day. Powdered bee bread and fermented pollen have been added to the honey in a ratio of 10% (*w*/*w*). The samples were then stored at 4 °C for three days and mixed mechanically twice a day. Analyses of creamed honeys with additives were carried out after their 60-day storage at ambient temperature.

### 3.4. Enriched Honey Analysis

#### 3.4.1. Physicochemical Parameters

Selected physicochemical parameters describing the quality of honey were determined: water content, electrical conductivity, pH, and free acidity. The determinations were made for 20% suspensions of honeys in deionized water as described earlier by Miłek et al. [46]. Energy value was determined using AC 500 calorimeter (LECO, St. Joseph, MI, USA) (oxygen bomb system) following ISO 18125:2017-07 [50].

The mineral content of enriched honey was calculated based on previously published data for honey [32], bee bread, and fermented pollen [17]. Assuming the additive nature of the enrichment, we estimated the final content of mineral ingredients in enriched honeys, taking into account the % share of the final product ingredients (90% honey and 10% additive).

#### 3.4.2. Total Phenolic Content and Antioxidant Properties

The analysis of the total content of phenolic compounds and antioxidant capacity was carried out for 20% honey solutions in deionized water and filtered through paper filters. The total phenolic content (TPC) using the Folin–Ciocalteu method and antioxidant capacity using the DPPH and FRAP methods were analyzed as described by Miłek et al. [46].

#### 3.4.3. Total and Soluble Protein Determination

The content of total nitrogen in tested samples was determined by the Kjeldahl method, in accordance with the PN-75/A-04018 standard: “Agricultural and food products. Determination of nitrogen by the Kjeldahl method and conversion to protein” [51]. Kjeltec apparatus (combustion and distillation set) (FOSS, Hilleroed, Denmark) was used for the study, taking into account the nitrogen to protein conversion factor. The sample was mineralized in concentrated sulfuric acid in the presence of a catalyst at a temperature of approx. 300 °C and the obtained content of titrated nitrogen was calculated from the Formula (1):

(1) $$X=\frac{0.01401\left(V\;\times {\;C}_{m}-{\;V}_{1}\times {\;C}_{m}\right)\times 100}{m}$$

where: X—grams of nitrogen corresponding to 100 g of the tested product, V—the volume (cm^3^) of the standardized HCl solution (0.1 mol/dm^3^), used for titration in the main sample, V_1—_the volume (cm^3^) of the standardized HCl used for titration in the blank sample, C_m_—the exact molar concentration of the HCl solution used for the titration, m—mass (g) of the tested product taken for the mineralization and then distillation, and 0.01401—the number of grams of nitrogen corresponding to 1 millimole of HCl or 1 cm^3^ of HCl solution with a concentration of strictly 1 mol/dm^3^.

The calculated nitrogen content was converted into protein using a conversion factor of 5.6 [52].

The soluble protein content in the samples was determined by the Bradford method according to Latimer [53]. The absorbance was read at 595 nm using a microplate reader (EPOCH 2, BioTek, Winooski, VT, USA). The results were calculated based on a calibration curve 1.8–250 μg/per sample (y = 0.0424x, R^2^ = 0.9658). Bovine albumin was used as a standard.

#### 3.4.4. SDS PAGE Protein Profile Analysis

Honey samples were prepared according to the procedure developed earlier [54]. Twenty microliters of dissolved honey were combined with 10 µL of 4× Laemmli buffer and after heat denaturation separated on 15% SDS-PAGE gels. Electrophoresis was performed in a Mini-Protean II apparatus (Bio-Rad Laboratories, Hercules, CA, USA) according to a standard procedure, using the BlueEasy Prestained Protein Marker ladder. After electrophoresis, the gels were stained as previously described [54] and analyzed in ImageJ 1.52a software.

#### 3.4.5. In Vitro Bioaccessibility of the Enriched Honeys

A study of the bioaccessibility of selected components in honeys enriched with natural and artificial bee bread was carried out according to Dżugan et al. [55]. Samples (2.5 g) of selected enriched honey (HBB2, HFP3, and HFP4) and control honey were subjected to static gastro-intestinal digestion in duplicate. After digestion, each digested sample was then centrifuged at 4100× *g* for 20 min (MPW-351R, MPW Med. Instruments, Warsaw, Poland), and soluble protein content was determined in supernatants (undigested fraction and intestinal fraction). The digestibility of protein has been calculated according to Equation (2):

(2) $$\mathrm{Digestibility}\;\%=\frac{\mathrm{total}\;\mathrm{protein}\;\mathrm{content}\;\mathrm{before}\;\mathrm{digestion}}{\mathrm{supernatant}\;\mathrm{protein}\;\mathrm{content}\;\mathrm{after}\;\mathrm{digestion}}\;\times \;100\;$$

### 3.5. Yeast Strain and Growth Conditions

The strain used in this study is a haploid wild-type yeast *Saccharomyces cerevisiae* strain BY4741 (MATa *his3Δ leu2Δ met15Δ ura3Δ;* Euroscarf, Germany) and *sod1Δ* mutant (MATa *his3Δ leu2Δ met15Δ ura3Δ YJR104C::kanMX4*; Euroscarf, Germany). Yeast cells were grown in a standard rich liquid YPD medium (1% Difco yeast extract, 1% yeast bactopeptone, and 2% glucose) on a rotary shaker at 150 rpm or on a solid YPD medium containing 2% agar. The experiments were carried out at 28 °C.

### 3.6. Kinetics of the Growth Assay

Yeast cells were grown in a standard liquid YPD medium without (control) or with tested honeys on a rotary shaker at 150 rpm, or on a solid YPD medium containing 2% agar. The experiments were performed at 28 °C. The growth was monitored using an Anthos 2010 type 17 550 microplate reader (Wals, Austria) at 600 nm by measurements at 2 h intervals for 12 h. Each experiment was repeated at least three times with similar outcomes.

### 3.7. Spot Tests

Yeast cultures were grown to a logarithmic phase (OD 600 nm between 0.8 and 1). The cells were then pre-incubated for 2 h at 28 °C with rotary shaking in honey solutions in a honey/YPD medium of the ratio 1:5. The cells were then washed three times with sterile distilled water. The pellet was suspended in YPD medium and the cells were treated with hydrogen peroxide (Sigma-Aldrich, St. Louis, MO, USA) at a final concentration of 2 mM. After this, the cells were washed twice in sterile distilled water and resuspended in 1 mL of sterile water, and serially diluted to different cellular concentrations as indicated (10^–1^ to 10^–4^). Five microliters of each cell suspension were spotted onto YPD agar plates. Growth was registered 48 h after incubation at 28 °C. Three independent tests have been conducted to confirm all phenotypes described in this report.

### 3.8. Cellular Localization of the Rad52-GFP Proteins

Honeys’ solutions were added to a yeast cell suspension (Rad52-GFP; Thermo Fisher Waltham, MA, USA) at a density of 1 × 10^6^ cells/mL, incubated at 28 °C with shaking for 2 h, then cells were washed twice in PBS and added zeocin at a final concentration of 2.5 μg/mL, and incubated an hour at 28 °C with shaking. Rad52-GFP foci were observed using BX-51 fluorescence microscope (1000× *g* magnification; λex = 488 nm, λem = 510 nm), equipped with a DP-72 digital camera and cellSens Dimension 4.1 software (Olympus, Shinjuku, Japan).

### 3.9. Statistical Analysis

All analyses were performed in triplicate, the results were presented as mean ± standard deviation. The results were subjected to one-way ANOVA and the significance of differences was determined based on Tukey’s test (*p* = 0.05). For tests in the yeast model, Dunnet’s test was used (*p* = 0.001, 0.01 and 0.05). All calculations were made using Statistica 13.3 software (StatSoft, Tulsa, OK, USA).

## 4. Conclusions

Laboratory-fermented bee pollen can be successfully used as an additive to honey, positively affecting its nutritional properties and bioactivity. The obtained enriched honey can be a good form of introducing bee pollen into the diet. The protein digestibility of this product was only slightly lower than in the case of natural bee bread-enriched honey which was demonstrated using simulated in vitro digestion. Multiplied enhanced antioxidant activity and polyphenol content in enriched honey resulted in the ability to reduce the effects of oxidative stress in yeast exposed to hydrogen peroxide. Moreover, all tested honeys showed genoprotective potential against yeast DNA damage induced by zeocin. All enriched honeys introduced to the culture media caused an inhibitory effect against yeast growth regardless of the strain used for testing. The obtained results confirm the benefits of combining honey with both bee bread and its laboratory-obtained substitute, however, the mechanism of such synergistic interaction requires further research. New product proposals seem to be promising dietary supplement but their nutritional value should be verified, optimally using animal feeding experiments.

## Figures and Tables

**Table 1:** Physicochemical parameters of tested honey samples.

Sample	Water Content [%]	Water Activity	Conductivity [mS/cm]	pH	Free Acidity [mval/kg]	Energy Value [kcal/100 g]
H	17.80 ± 0.00 ^a^	0.5676 ± 0.0006 ^a^	0.149 ± 0.000 ^a^	4.25 ± 0.01 ^a^	8.95 ± 0.35 ^a^	330.6 ± 2.3 ^a^
HBB1	17.40 ± 0.00 ^b^	0.5712 ± 0.0025 ^b^	0.528 ± 0.001 ^b^	3.97 ± 0.00 ^b^	99.26 ± 1.07 ^b^	342.7 ± 3.1 ^bc^
HBB2	16.90 ± 0.28 ^c^	0.5673 ± 0.0000 ^a^	0.537 ± 0.000 ^c^	3.96 ± 0.01 ^c^	97.25 ± 2.47 ^b^	339.0 ± 3.5 ^bc^
HFP1	17.15 ± 0.21 ^bc^	0.5566 ± 0.0005 ^c^	0.424 ± 0.000 ^d^	4.35 ± 0.01 ^d^	65.85 ± 0.21 ^c^	340.5 ± 4.0 ^bc^
HFP2	17.85 ± 0.07 ^a^	0.5708 ± 0.0020 ^b^	0.426 ± 0.002 ^d^	4.38 ± 0.01 ^e^	62.95 ± 0.35 ^d^	338.2 ± 1.8 ^ab^
HFP3	16.95 ± 0.07 ^c^	0.5729 ± 0.0020 ^b^	0.459 ± 0.001 ^e^	4.48 ± 0.00 ^f^	62.20 ± 0.28 ^d^	341.1 ± 1.3 ^bc^
HFP4	15.75 ± 0.35 ^d^	0.5531 ± 0.0005 ^d^	0.601 ± 0.001 ^f^	4.15 ± 0.00 ^g^	90.00 ± 0.71 ^e^	347.0 ± 4.0 ^c^
HFP5	16.45 ± 0.07 ^e^	0.5569 ± 0.0000 ^c^	0.665 ± 0.001 ^g^	4.41 ± 0.01 ^h^	70.45 ± 0.49 ^f^	345.6 ± 2.4 ^bc^

^a–h^—the means marked with different letters in the columns differ statistically significantly (*p* < 0.05). H—control rapeseed honey, HBB1, HBB2—honey enriched with two samples of natural bee bread, HFP1-HFP5—honey enriched with laboratory-fermented bee pollen FP1-FP5 (symbols as described in Materials and Methods Section 3.2.).

**Table 2:** Total phenolic content (TPC) and antioxidant properties (FRAP, DPPH) of tested honeys.

Sample	TPC [mg GAE/100 g]	FRAP [μmol TE/100 g]	DPPH [μmol TE/100 g]
H	17.51 ± 0.90 ^a^	18.87 ± 0.94 ^a^	3.84 ± 5.28 ^a^
HBB1	80.43 ± 9.56 ^b^	105.15 ± 8.92 ^bc^	41.76 ± 1.95 ^b^
HBB2	88.91 ± 8.43 ^b^	133.00 ± 13.10 ^c^	46.07 ± 7.08 ^bc^
HFP1	81.67 ± 7.06 ^b^	117.71 ± 6.59 ^bc^	44.71 ± 2.85 ^bc^
HFP2	82.76 ± 10.39 ^b^	97.92 ± 7.46 ^b^	53.40 ± 11.66 ^bc^
HFP3	97.89 ± 19.05 ^bc^	111.57 ± 13.71 ^bc^	55.64 ± 10.77 ^bc^
HFP4	119.77 ± 9.60 ^c^	124.40 ± 13.73 ^bc^	65.21 ± 0.53 ^bc^
HFP5	101.64 ± 10.59 ^bc^	122.37 ± 9.18 ^bc^	68.40 ± 16.66 ^c^

^a–c^—the means marked with different letters in the columns differ statistically significantly (*p* < 0.05). H—control rapeseed honey, HBB1, HBB2—honey enriched with two samples of natural bee bread, HFP1-HFP5—honey enriched with laboratory fermented bee pollen FP1-FP5 (symbols as described in Materials and Methods Section 3.2.).

**Table 3:** Protein content in tested samples.

Sample	Total Protein (Kjeldahl Method) [g/100 g]	Soluble Protein (Bradford Method) [g/100 g]
H	0.220 ± 0.006 ^a^	0.174 ± 0.004 ^a^
HBB1	2.207 ± 0.026 ^b^	0.858 ± 0.009 ^b^
HBB2	2.107 ± 0.041 ^b^	0.818 ± 0.021 ^bc^
HFP1	1.633 ± 0.067 ^cd^	0.863 ± 0.027 ^b^
HFP2	1.707 ± 0.056 ^c^	0.782 ± 0.038 ^c^
HFP3	1.692 ± 0.035 ^cd^	0.714 ± 0.010 ^d^
HFP4	1.729 ± 0.050 ^d^	0.494 ± 0.036 ^e^
HFP5	1.698 ± 0.008 ^cd^	0.616 ± 0.022 ^f^

^a–f^—the means marked with different letters in the columns differ statistically significantly (*p* < 0.05). H—control rapeseed honey, HBB1, HBB2—honey enriched with two samples of natural bee bread, HFP1-HFP5—honey enriched with laboratory-fermented bee pollen FP1-FP5 (symbols as described in Materials and Methods Section 3.2.).

**Table 4:** A composition of laboratory-fermented pollen samples.

Sample	Bee Pollen	Multifloral Honey	Water	Starter Culture	Fermentation Time and Temperature
FP1	10 g	7.5 g	12.5 mL	*L. rhamnosus* GG 1 g	48 h in 32 °C and 4 weeks in 25 °C
FP2	10 gultrasound treated (2 × 15 min., 700 W)	7.5 g	12.5 mL	*L. rhamnosus* GG 1 g	48 h in 32 °C and 4 weeks in 25 °C
FP3	10 g	7.5 g	12.5 mL	-	48 h in 32 °C and 4 weeks in 25 °C
FP4	10 g	3.33 mL (60% solution)	-	*L. acidophilus* 1 g	120 h in 37 °C
FP5	10 g	3.33 mL (60% solution)	10 mL	*L. acidophilus* 1 g	120 h in 37 °C

## Data Availability

The data presented in this study is available in the article.

## Sample Availability

Samples are no longer available from the authors.
